# Quality of Life After Transradial Access in Cerebral Angiography: A SF-12 Analysis Using a Then-Test Design

**DOI:** 10.3390/healthcare13131509

**Published:** 2025-06-24

**Authors:** Johannes Rosskopf, Julian Kifmann, Bernd Schmitz, Michael Braun

**Affiliations:** 1 Section of Neuroradiology, Bezirkskrankenhaus Guenzburg, 89312 Guenzburg, Germany; 2 Department of Diagnostic and Interventional Radiology, University Hospital Ulm, 89081 Ulm, Germany; 3 Department of Oral and Plastic Maxillofacial Surgery, Armed Forces Hospital Ulm, 89081 Ulm, Germany

**Keywords:** health-related quality of life, quality of life, 12-item short form health survey, SF-12, physical component summary, PCS, mental component summary, MCS, transradial cerebral diagnostic catheter angiography

## Abstract

**Background**: Transradial access may affect health-related quality of life (QoL) in cerebral diagnostic angiography. However, its assessment is methodologically challenging, as repeated measurements can be influenced by response shift. To mitigate this bias, a retrospective cross-sectional study was designed using a then-test approach, allowing patients to reflect on their post procedural status at a single time point. **Methods**: Quality of life was assessed using the 12-Item Short Form Health Survey (SF-12). A then-test approach was also employed, whereby patients were asked to retrospectively indicate whether they perceived their condition as worse following the procedure. The survey yielded Physical (PCS) and Mental Component Summary (MCS) scores, standardized to a mean of 50 (range of 0–100), with lower values indicating greater health-related limitations. Group differences were analyzed using the Mann–Whitney U test. Associations between PCS and MCS, respectively, and clinical variables were assessed using multiple linear regression models. **Results**: Forty patients underwent diagnostic cerebral angiography over a 15-month observation period. Applying a then-test design, Group A included the 12.5% (n = 5) of patients who reported feeling worse post-procedure while Group B comprised the remaining 87.5% (n = 35). QoL scores were significantly lower in Group A (Mdn = 28.6) compared to B (Mdn = 46.7) for both PCS scores (*p* = 0.007) and MCS scores (45.3 vs. 54.6, *p* = 0.018). In the multiple linear regression analysis, no statistically significant associations were found between the PCS or MCS scores and any clinical variable, including age, sex, body mass index (BMI), procedure duration, dose area product, access site, prior neurosurgical history, and fluoroscopy time (*p* > 0.05). **Conclusions**: Transradial access for diagnostic cerebral angiography may affect QoL, as assessed using the SF-12 questionnaire. Applying the then-test approach, the group of patients who reported feeling worse after the procedure (12.5%) showed significantly lower physical and mental health scores. These findings underscore the need for prospective studies to further investigate patient-reported outcomes.

## 1. Introduction

The transradial approach is increasingly adopted in neurointervention and has emerged as the access route most preferred by patients undergoing catheter-based angiography [1,2,3,4,5]. Its lower rates of access-site bleeding, major vascular complications, and adverse clinical events compared to the transfemoral approach [6,7] support its safety.

Despite its clinical advantages and even though its adoption is rising, transradial access remains far from being standard practice in cerebral angiography. One possible reason might be its influence on health-related quality of Life (QoL). In summary, the few available studies in cardiology have suggested that QoL was either not significantly affected or even improved when using the transradial approach compared to the transfemoral approach in diagnostic cardiac catheterization or in the endovascular treatment of ST-segment elevation myocardial infarction [8,9,10,11,12].

Nevertheless, in the context of cerebral angiography, the transradial approach may have a distinct impact on QoL in cerebral angiography due to procedural discomfort, patient awareness, and the ambulatory setting. Unlike procedures performed under general anesthesia, patients undergoing diagnostic cerebral angiography via radial access are fully conscious, which may heighten psychological stress—particularly in technically more complex or anxiety-provoking cerebral angiography [1,13]. Even minor postprocedural symptoms, such as wrist pain or fatigue, could affect a patient’s perception of well-being, especially when early discharge and rapid return to self-care are expected [10]. Furthermore, while transradial access is generally safe, transient vascular discomfort or temporary hand dysfunction may occur [14,15,16]. Collectively, these aspects could subtly, but measurably, influence both physical and mental dimensions of quality of life. 

Health-related QoL can be assessed using various standardized instruments. The 12-Item Short Form Health Survey (SF-12) [17,18] is a widely used short-form questionnaire that captures both physical and mental health status through two composite scores: the Physical Component Summary (PCS) and the Mental Component Summary (MCS). Compared to the EuroQol 5-Dimensions Questionnaire (EQ-5D) [19], which is commonly used in health economic evaluations due to its ability to generate utility values for quality-adjusted life year calculations [20], the SF-12 covers a broader range of health dimensions and offers greater sensitivity to subtle changes in functional or psychological status. Its multidimensional structure makes it suitable for clinical research contexts where broader profiling of physical and mental health is required beyond utility-based measures. For these reasons, the SF-12 was selected to capture a more nuanced picture of patient-reported outcomes following diagnostic cerebral angiography, where only subtle changes in physical and mental health were expected.

Methodologically, QoL assessment is challenging due to potential response bias, such as response shift in repeated measurements [21]. One established solution is the then-test approach, [22] in which patients retrospectively compare their pre- and post-intervention health status from their current perspective. In the context of this retrospective study, the then-test provided a straightforward and appropriate method for capturing perceived changes in physical and mental health.

This study aimed to investigate the effect of the transradial access on QoL which has not yet been systematically analyzed in the context of cerebral diagnostic catheter procedures.

## 2. Materials and Methods

### 2.1. Study Design and Patient Selection

Diagnostic cerebral catheter angiographies at our institution are primarily performed via a transradial approach. For this retrospective observational study, all patients undergoing transradial procedures between January 2020 and March 2021 were identified from the institutional database. A 12-month period was estimated to allow for reliable recall of the angiographic experience. The actual data collection period extended to 15 months, primarily due to organizational factors. Patients with crossover to femoral access were not excluded. The local ethics committee approved the study (reference #238/21), and the need for informed consent was waived due to its retrospective design. All patient data were anonymized before analysis.

### 2.2. Transradial Approach

With the forearm positioned in full supination and secured using an angiography arm board, radial artery access was obtained under sterile conditions. Approximately 10 mL of local anesthetic (Prilocaine hydrochloride 10 mg/mL, Xylonest^®^ 1%, Aspen Germany GmBH, Munchen, Germany) was administered subcutaneously to minimize procedural pain and reduce the risk of vasospasm.

Radial puncture was performed by palpation using a 20-gauge needle, followed by the insertion of a 5-French Glidesheath Slender^®^ (Terumo Germany GmBH, Eschborn, Germany) via the Seldinger technique. Intra-arterial administration of verapamil (2.5 mg) and heparin (5000 IU) over 2–3 min was used to prevent vasospasm and thrombosis while minimizing hypotensive effects. Target vessels were selectively catheterized using a 0.035″ guidewire and a diagnostic catheter, most commonly a 5-French Simmons-2. Continuous flushing of the diagnostic catheter with heparinized saline was maintained throughout the procedure.

Upon completion of angiography, hemostasis was achieved using a TR BAND^®^ radial compression device (Terumo Germany GmBH, Eschborn, Germany), allowing gradual air titration to ensure patent hemostasis. The device was slowly deflated over two hours and subsequently removed. The patients remained ambulatory during this period.

Neither preprocedural assessment of collateral circulation (e.g., Allen’s or Barbeau test) nor routine ultrasound guidance for vascular access was performed.

### 2.3. Telephone Interview

In accordance with the requirements of the local ethics committee, written informed consent was obtained from all patients prior to telephone contact. The purpose of the telephone interview was to assess health-related QoL after angiography using the self-reported SF-12 questionnaire. In addition, a then-test question was included, asking patients whether they perceived their health status as worse compared to before the procedure.

The SF-12 is a validated, generic instrument for measuring health-related quality of life [17,18]. It condenses the eight domains of the longer SF-36 (physical functioning, role-physical, bodily pain, general health, vitality, social functioning, role-emotional and mental health) into 12 items, which are weighted and aggregated to produce two composite indices, the PCS and MCS. The scores are norm-based, standardized to a population mean ± SD of 50 ± 10, and expressed on a 0–100 scale; lower values reflect greater health limitations, while higher values indicate better perceived health.

For this study, the SF-12 was administered by trained research staff during a structured telephone interview. The interviewer read each item verbatim from a scripted questionnaire and recorded the patient’s response in real time.

### 2.4. Statistical Analysis

All statistical analyses were performed using SPSS (version 30; IBM Corp., Armonk, NY, USA). Demographic and clinical variables were reported as means with standard deviations (SDs).

Since the PCS and MCS scores were not normally distributed, group comparisons were conducted using the Mann–Whitney U test to evaluate differences between Group A (patients who reported feeling worse post-procedure) and Group B (all other patients). Multiple linear regression analyses were performed to assess potential associations between the QoL scores and variables, including age, sex, body mass index (BMI), procedure duration, dose area product (DAP), access site, prior neurosurgical history, and fluoroscopy time. A two-sided *p*-value of <0.05 was considered statistically significant.

## 3. Results

### 3.1. Study Sample

During the 15-month observation period from 2020 to 2021, a total of 88 patients were enrolled. In two cases, crossover to transfemoral access was necessary due to failed radial access. These patients did not respond to the written invitation and were, therefore, not included in the final cohort. Additionally, two patients underwent cerebral angiography twice during the study period. Only the first procedure was included in the analysis to avoid duplicate data.

Of the remaining patients, 40 patients (45%) completed a structured telephone interview. In this cohort, no patient required crossover to femoral access, and procedural success was 100% in this cohort. All data points were available for analysis.

Based on the then-test design, patients were divided into two groups according to their retrospective self-assessment of health status following the procedure. Group A comprised 12.5% of participants (n = 5) who reported feeling worse post-procedure, while Group B included the remaining 87.5% (n = 35) who reported stable or improved health. Cerebral catheter angiography was performed postoperatively in 80% of cases, with right-sided radial access used in 85%. In eight cases, angiography was performed using a vertebral-configured diagnostic catheter; in the remaining cases, a SIM-2 catheter was used. Arterial patency was assessed post-procedurally by palpation of the radial pulse. Vasospasm was observed in four patients (10%). No cases of radial artery occlusion or pseudoaneurysm were detected. Further demographic and procedural data, stratified by Group A and Group B, are summarized in Table 1.

### 3.2. Follow-Up Imaging Findings

Post-treatment angiography was performed to assess aneurysm status (n = 28), as well as after treatment of dural arteriovenous fistulas, arteriovenous malformations, and following bypass surgery in cases of moyamoya disease. Aneurysm recurrence was detected in four cases of previously ruptured, endovascularly treated aneurysms (Table 2).

Diagnostic cerebral angiography without surgery before was performed in six cases to evaluate dural arteriovenous fistula. In one of these cases, the presence of a fistula was not confirmed. Additionally, diagnostic angiography was conducted in one case to analyze a complex internal carotid artery aneurysm, and in another case to assess the status of a moyamoya malformation using digital subtraction angiography.

### 3.3. Quality of Life

**Figure 1:**
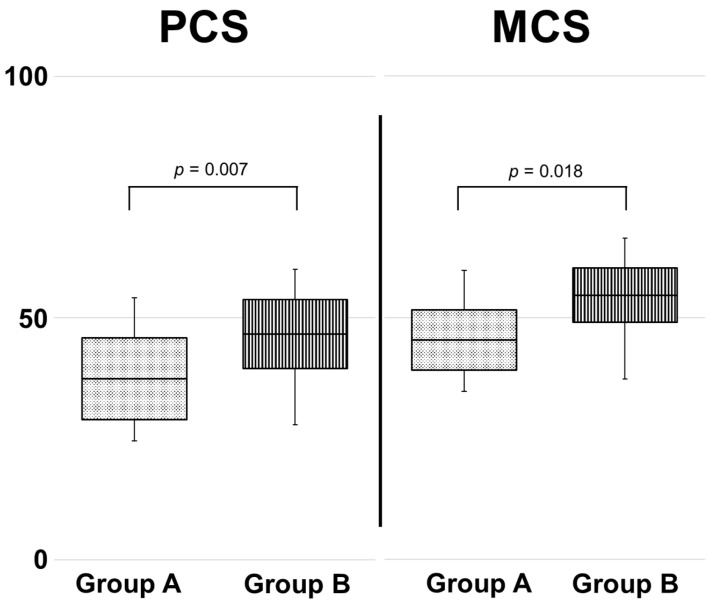
Comparison of SF-12 physical and mental component scores between Group A and Group B. Quality of life scores were significantly lower in Group A (feeling worse post-procedure) compared to B for both PCS and MCS. Results are displayed as box plots.

The comparison of health-related QoL between Groups A and B revealed significantly lower scores in Group A (feeling worse post-procedure) for both the Physical Component Summary (PCS) and the Mental Component Summary (MCS) of the SF-12 (Figure 1). Specifically, the median PCS scores were 28.6 in Group A and 46.7 in Group B, U = 24, z = −2.6, *p* = 0.007, r = −0.41. The median MCS scores were 45.3 and 54.6, respectively, U = 31, Z = −2.311, *p* = 0.018, r = −0.37. According to Cohen (1992) [23], these differences represent medium effect sizes.

### 3.4. Correlation Analyses

No statistically significant associations were found between the QoL scores (PCS or MCS) and other variables, including age, sex, body mass index (BMI), procedure duration, dose area product, access site, prior neurosurgical history, and fluoroscopy time (Table 3). For BMI, we excluded one extreme outlier (BMI = 54), resulting in a total of 39 cases for the association analysis. Since the DAP, total duration, and fluoroscopy time were not normally distributed (*p* < 0.001 for all), these variables were log-transformed (natural logarithm), which resulted in normal distributions. For the PCS model, the adjusted R^2^ was 0.018, F(9, 29) = 1.120, *p* = 0.381. For MCS, the adjusted R^2^ was 0.174, F(9, 29) = 1.892, *p* = 0.094.

## 4. Discussion

Quality of life after transradial diagnostic cerebral catheter angiography was assessed using the SF-12 questionnaire. Applying a then-test approach, group comparisons showed significantly lower PCS and MCS scores in patients reported feeling worse after the procedure. No significant associations were found for age, sex, BMI, procedure duration, dose area product, access site, prior neurosurgical history, and fluoroscopy time.

The overall QoL in patients undergoing neurointerventions tends to be lower than in the general population [24,25]. In 2020, Drixler et al. [18] reported normative SF-12 scores from a representative German sample (n = 2524), with mean PCS and MCS values of 54.36 ± 10 and 53.5 ± 10, respectively. So, Pala et al. [24] compared SF-36 scores of 79 patients after treatment of unruptured intracranial aneurysms with normative data and found reduced QoL, with mean PCS and MCS scores of 43.7 and 45.1. In the present study, using a then-test approach, patients in Group A who reported feeling worse post-procedure showed even lower PCS and MCS scores (32.6 and 42.3) compared to Pala et al. [24], suggesting that the transradial catheter approach may negatively impact quality of life in a subset of patients.

In the cardiology literature, Cooper et al. [26] were among the first to investigate QoL after transradial access in cardiac catheterization. In their 1999 study of 101 patients, they found that transradial access was associated with improved QoL compared to transfemoral access one week post-procedure, as measured by the SF-36. Subsequent studies supported these findings. In patients with ST-segment elevation myocardial infarction, QoL improvements were confirmed in follow-up assessments using the EQ-5D questionnaire [9], and in another study, radial access was associated with the lowest level of discomfort compared to femoral access based on visual analog scales [27]. Reddy et al. [10] later reported no significant difference in SF-36 scores between radial and femoral access in the context of rapid ambulation one hour after cardiac catheterization. Most recently, in 2021, the brachial approach was linked to more frequent self-care difficulties compared to radial and femoral access following invasive cardiology procedures [12]. In the current study, the use of a then-test design may have enhanced sensitivity for detecting subtle changes in self-reported health status. Nevertheless, our findings are compatible with previous research, as the majority of patients (87.5%) did not report a lower QoL on PCS or MCS compared to the values reported by, e.g., Pala et al. [24] in patients following treatment of unruptured intracranial aneurysms.

No statistically significant associations were found between the QoL scores (PCS or MCS) and other variables. This is in line with the findings of previous studies. The aforementioned studies in cardiology did not report any association between sex and QoL. Furthermore, in a randomized trial evaluating access site strategy in female patients using the EQ-5D, no difference in QoL was found between the radial and femoral arteriotomy groups [8].

This study has several limitations. Its retrospective design precluded the collection of baseline QoL scores prior to the procedure. However, given the aim of the study to provide a general assessment of the impact of elective transradial access for diagnostic cerebral angiography on QoL, the then-test approach was deemed appropriate. However, the use of the then-test entails recall bias and the inherent subjectivity of retrospective self-assessment, which represent an additional limitation and should be taken into account when interpreting the results. Furthermore, no comparison with a transfemoral cohort was possible, as the transradial approach is the standard access route for diagnostic cerebral catheter angiography at our institution, and an adequate number of transfemoral cases was not available during the study period. While the learning curve for radial access is known to be steep [13], the absence of a transfemoral control group also reflects our institution’s high level of experience with the transradial technique. In addition, only 12.5% of patients (n = 5) were included in Group A (those reporting worsened QoL post-procedure), and a larger subgroup size would have been desirable. Therefore, Group A did not allow for a representative reflection of the overall study population. Also, the relatively short procedure time and narrow time range in our cohort may have contributed to the low variance, thus limiting the detectability of associations with the time variables. Given the brief nature of the angiographic procedure, the absence of associations may also reflect a ceiling effect in patients already near baseline health. Moreover, radial access in this study was exclusively obtained via the conventional volar approach. Therefore, potential effects on quality of life associated with the distal approach via the anatomical snuffbox, which has been shown to be a safe and effective alternative [28], could not be assessed. Future prospective studies on the transradial approach are needed to address these limitations and further validate the findings.

## 5. Conclusions

Transradial access for diagnostic cerebral catheter procedures appears to impact health-related QoL, as assessed using the SF-12 questionnaire. Applying the then-test approach, subtle changes in patient-reported outcomes were identified. Notably, a group of patients (12.5%) retrospectively reported feeling worse after the procedure and exhibited significantly lower scores in both physical and mental health domains. This highlights the importance of capturing individual perceptions through retrospective self-assessment methods, especially in minimally invasive and outpatient settings. Nevertheless, the majority of patients did not report any deterioration. Larger prospective studies are warranted to further evaluate patient-reported outcomes.

## Figures and Tables

**Table 1:** Demographic and procedural data.

	Group A (n = 5)	Group B (n = 35)
**Age** (years)	58.8 ± 11.4	56.9 ± 12.8
**Sex** (m/f)	1/4	14/21
**Angiography before/after treatment**	0/5	8/27
**Vasospasm** (yes/no)	2/3	3/33
**Radial access site** (right/left)	5/0	29/6
**BMI** (kg/m^2^)	29.3 ± 6.5	26.6 ± 6.4
**DAP** (µGym^2^)	3858 ± 1739.0	5245.2 ± 5044.0
**Total duration** (minutes)	21.8 ± 11.8	23.2 ± 15.1
**Fluoroscopy time** (minutes)	9.3 ± 3.7	10.8 ± 9.0

Group A comprised patients who reported feeling worse post-procedure, and Group B comprised the remaining patients. Mean ± standard deviation; BMI: body mass index; DAP: dose area product.

**Table 2:** Imaging findings from post-treatment angiography.

	Aneurysm		dAVF	AVM	Bypass
	Ruptured	Unruptured			
**Endovascular-treated**	22 (4)	3 (0)	1 (0)		
**Surgery-treated**	3 (0)			2 (0)	1 (0)

Distribution of diagnostic objectives for angiography in patients with prior surgery. The numbers in brackets indicate the number of cases with relevant findings requiring additional treatment. dAVF: dural arteriovenous fistula; AVM: arteriovenous malformation; Bypass: bypass surgery.

**Table 3:** Multiple linear regression models predicting physical (PCS) and mental (MCS) health summary scores.

	PCS			MCS		
	β	95%-CI	*p*-Value	β	95%-CI	*p*-Value
**Age**	−0.180	(−0.434; 0.146)	0.32	0.286	(−0.036; 0.461)	0.09
**Sex**	0.120	(−6.766; 11.685)	0.59	0.378	(−0.687; 15.120)	0.07
**Angiography before/after**	0.303	(−4.077; 19.657)	0.19	0.128	(−7.123; 13.211)	0.55
**Vasospasm**	−0.234	(−10.002; 2.549)	0.23	−0.257	(−9.181; 1.571)	0.16
**Radial access site**	0.040	(−10.007; 12.217)	0.84	−0.044	(−10.631; 8.409)	0.81
**BMI**	−0.245	(−1.343; 0.294)	0.20	−0.018	(−0.738; 0.665)	0.92
**DAP** *	−0.057	(−8.179; 6.427)	0.81	−0.409	(−12.109; 0.405)	0.07
**Total duration** *	−0.077	(−10.049; 7.237)	0.74	−0.216	(−11.065; 3.744)	0.32
**Fluoroscopy time** *	0.033	(−7.436; 8.415)	0.90	0.439	(−0.688; 12.893)	0.08

Values represent regression coefficients (β) with 95% confidence intervals (CI) and *p*-values. BMI: body mass index; DAP: dose area product; PCS: physical component summary; MCS: mental component summary; * DAP, total duration, and fluoroscopy time were not normally distributed and, therefore, log-transformed, which resulted in normal distributions.

## Data Availability

Data are contained within the article.

## Abbreviations

The following abbreviations are used in this manuscript:

QoL	Quality of Life
SF-12	12-Item Short Form Health Survey
PCS	Physical Component Summary
MCS	Mental Component Summary
BMI	Body Mass Index
EQ-5D	EuroQol 5-Dimensions Questionnaire
SD	Standard Deviation
SPSS	Statistical Package for the Social Sciences
